# Use of gain-of-function suppressor screening to elucidate *Candida albicans* Cdr1 efflux mechanism, inhibition, and drug resistance

**DOI:** 10.3389/fmicb.2025.1721050

**Published:** 2025-12-10

**Authors:** Mengcun Zhao, Masakazu Niimi, Richard D. Cannon, Erwin Lamping

**Affiliations:** Sir John Walsh Research Institute, Faculty of Dentistry, University of Otago, Dunedin, New Zealand

**Keywords:** *Candida albicans*, Cdr1, efflux pump, PDR transporter, ABC transporter, inhibitor, gain-of-function, suppressor screening

## Abstract

The ATP-binding cassette (ABC) transporter Cdr1 is a key mediator of antifungal drug resistance in *Candida albicans*, a major pathogen responsible for invasive candidiasis. Cdr1 belongs to the pleiotropic drug resistance (PDR) ABC transporter subfamily, exhibits broad substrate specificity, and plays a central role in protecting *C. albicans* cells from azole drugs and many other xenobiotics. Although high-resolution structures of Cdr1 and its homologs are now available, the transport cycle of Cdr1 remains incompletely understood. Furthermore, although these structures provide valuable information about the spatial arrangements of amino acids, they do not reveal how, and which, amino acid interactions affect pump function and how to relieve drug resistance. Drug resistance–based gain-of-function suppressor screening has emerged as a powerful tool to identify inhibitor binding hotspots, and conformational adaptation mechanisms. These studies reveal residues critically important for the structural and functional integrity of Cdr1, offering deeper mechanistic insights into substrate/inhibitor recognition and transport. Importantly, these insights will aid in the rational development of Cdr1 inhibitors that, when combined with azoles, may reverse efflux pump-mediated drug resistance. Inhibitors that stabilize discrete conformations of Cdr1 offer additional tools for the structural and mechanistic characterization of this important efflux pump. Here, we demonstrate the power of gain-of-function suppressor screening for determining functionally important residues and validating structural models of the prototype fungal PDR transporter, *C. albicans* Cdr1.

## 1 Introduction

*Candida* species are common commensals in healthy individuals (Sardi et al., 2013). However, many are opportunistic pathogens capable of causing superficial infections and, under certain conditions, life-threatening invasive candidiasis, especially in immunocompromised individuals. Invasive candidiasis accounts for 70–90% of ICU-associated fungal infections (Noppè et al., 2024) with a 30-day mortality rate of 40–55% despite antifungal treatment (Soriano et al., 2023). Among *Candida* species, *Candida albicans* remains the most frequently isolated species in invasive candidiasis globally (Pappas et al., 2016; Pfaller et al., 2019).

A major challenge in treating invasive candidiasis is the emergence of drug-resistant strains. One key factor contributing to this is the overexpression of drug efflux pumps (Lee et al., 2021). *C. albicans* Cdr1 is an important ATP-binding cassette (ABC) transporter that expels from cells a broad range of antifungals, including the widely used azoles. Cdr1 is a type V ABC transporter (Thomas et al., 2020) and a member of the ABCG subfamily (Dean et al., 2001; Paumi et al., 2009), which in fungi and plants are commonly known as pleiotropic drug resistance (PDR) transporters (Verrier et al., 2008; Lamping et al., 2010). Full-length PDR transporters feature a topology with each of the two nucleotide-binding domains (NBDs) preceding a transmembrane domain (TMD), forming a [NBD-TMD]_2_ arrangement (Lamping et al., 2010; Thomas et al., 2020). A promising approach to overcome multidrug resistance mediated by fungal PDR transporters is to identify or develop effective inhibitors of these efflux pumps. This requires a precise understanding of their transport mechanisms and modes of inhibition.

Following the solving of the human sterol transporter ABCG5/G8 structure (Lee et al., 2016), a number of ABCG transporter structures in various conformational states have been reported (Lee et al., 2016; Taylor et al., 2017; Manolaridis et al., 2018) including *Saccharomyces cerevisiae* Pdr5 (Harris et al., 2021), and more recently Cdr1 itself (Peng et al., 2024). These have provided important snapshots of the transport cycle but failed to provide evidence for the role of amino acid interactions in pump function and inhibitor resistance.

To investigate the roles of key residues in these processes, both site-directed mutagenesis and various random mutagenesis approaches – such as UV-, hydroxylamine-, or error-prone PCR-induced mutagenesis – have been widely employed. A limitation of site-directed mutagenesis is that it is only as good as the investigator’s hypothesis in terms of deciding which residue to modify in what particular way. It suffers, therefore, from investigator bias. Although random mutagenesis is an unbiased approach, often the procedures generate multiple mutations which can be difficult and/or time consuming to decouple. In this article we provide our perspective on the value of gain-of-function drug resistance–based suppressor screening to identify compensatory mutations that restore function in an unbiased and relatively fast manner. We highlight saturation suppressor screening as a particularly powerful and broadly applicable tool for elucidating transporter functions.

## 2 Cdr1 structure and proposed transport model

ABC transporters feature conserved NBDs for ATP hydrolysis, but more divergent TMDs, reflecting their need to accommodate diverse pump substrates. The typical transport cycle model involves substrate entry, followed by ATP binding–induced NBD dimerization, which drives TMD movement towards an outward-facing conformation and substrate release. Subsequent ATP hydrolysis and the release of phosphate and ADP reset the transporter.

**Figure 1:**
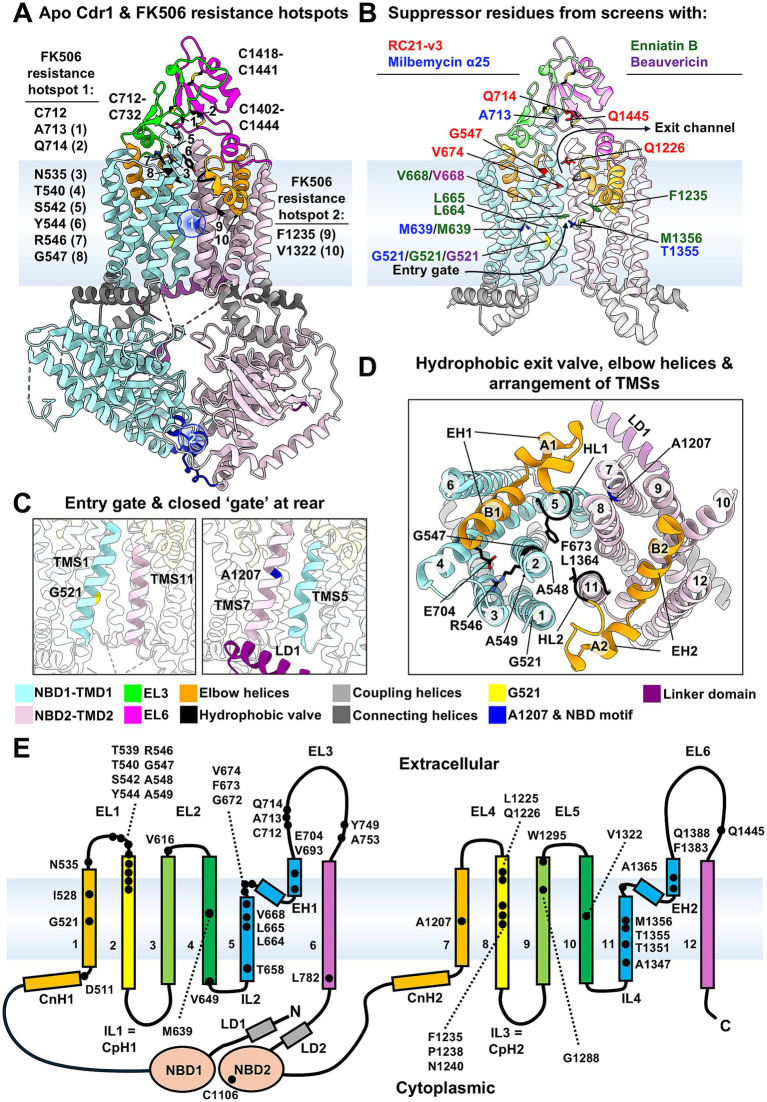
**(A–D)** Cartoon models of the apo-state cryo-EM structure of Cdr1 (Protein Data Bank entry code 9IUK, Peng et al., 2024). The color indicators are shown underneath panels **(C,D)**. **(A)** Location of the two pivotal contact points (blue circles): (1) TMS5-TMS7, with key contact residue A1207 in TMS7; and (2) GX_2[3]_CPX_3_NPXD/E motifs, key contacts between NBD1 and NBD2 at the cytosolic apex. The figure also indicates the FK506 resistance hotspot 1 and hotspot 2 residues in Cdr1. Disulfide bonds and FK506 resistance suppressor residues are shown as sticks (O, red; N, blue; S, yellow). **(B)** The distribution of suppressor residues (shown as colored sticks) conferring resistance of fluconazole transport to RC21-v3 (red), milbemycin α25 (blue), enniatin B (green) and beauvericin (purple). NBDs and the linker domain (LD1 and LD2) at the rear of the TMDs are omitted for clarity of view, and the cartoon model is half transparent. **(C)** The entry gate at G521 (left) and LD1 promoting a permanently closed “gate” at the rear (right) of the transporter with the key A1207 contact residue. **(D)** Close-up, top-down, view of the TMDs with the 12 TMSs highlighting the hydrophobic exit valve and the two hydrophobic loops, HL1 and HL2, at the centre of the transporter surrounded by two elbow helices, EH1 and EH2, on either side of the substrate exit channel. The side chains of A1207 in TMS7, the hydrophobic exit valve residues F673 and L1364, the RGAA motif residues R546, G547, A548, A549, and the conserved E704 at the C-terminus of EH1 are shown as sticks. The models were visualized with UCSF ChimeraX (Meng et al., 2023). **(E)** Topology of Cdr1 and the location of the 48 inhibitor-resistant and secondary site suppressor residues that were isolated on 121 separate occasions are shown as black dots. EL: extracellular loop; IL: intracellular loop. The plasma membrane bilayer in **(A,B,E)** are represented by a light blue background.

A hallmark of full-length PDR transporters, including Cdr1, is their structural asymmetry (Lamping et al., 2010; Gupta et al., 2011). Only one of the two nucleotide-binding sites (NBS2) is catalytically active; NBS1 binds but does not hydrolyze ATP due to degeneration in key conserved NBD motifs (Walker A1, Walker B1, ABC signature2, Q-loop1, and H-loop1). ATP is thought to remain bound to NBS1 throughout the transport cycle, stabilized by interactions with linker domain 1 (LD1) and LD2 (Figures 1A,C) (Harris et al., 2021).

The two TMDs each comprise six transmembrane segments (TMSs). The central substrate-binding pocket is formed by TMS1, −2, −5, −8 and −11 (Figure 1D). Entry from the lipid bilayer is gated by the flexible TMS1 (facing TMS11 and located above the catalytically active NBS2; Figures 1B,C). The hydrophobic valve – primarily involving F673 (TMS5) and L1364 (TMS11) (Figure 1D) – is another conserved feature of ABCG transporters, located above the binding pocket (Figure 1B) and forming a one-way molecular diode (Gupta et al., 2011) preventing drug reflux (Khunweeraphong et al., 2019; Alhumaidi et al., 2022).

Cdr1 also features a large extracellular domain (ECD) consisting of four short and two long extracellular loops (ELs), stabilized by three conserved disulfide bonds (C712–C732, C1402–C1444, C1418–C1441; Figure 1A). The ECD’s precise role remains unclear, but it certainly contributes to protein folding and stability, and drug efflux (Madani et al., 2021).

TMS1 and TMS7 are each preceded by a connecting helix (CnH) that aligns parallel to the inner leaflet of the lipid bilayer. These two CnHs and the two coupling helices (CpHs) of the large intracellular loops (ILs) IL1 and IL3 between TMS2/3 and TMS8/9 (Figures 1A,B), respectively, play an important role in converting rigid body motions at the NBDs into large conformational changes of the TMDs during substrate transport (Khunweeraphong et al., 2017; Vishwakarma et al., 2019). PDR transporters also feature conserved elbow helices (EHs) surrounding the hydrophobic exit valve at the extracellular water-lipid bilayer interface. EH1 and EH2 comprise two short alpha helices separated by conserved proline-linkers (Lamping et al., 2010) that serve as the elbows connecting the two “upper arms” (i.e., PDR A1 and A2 helices) with the two “forearms” (i.e., PDR B1 and B2 helices) of Cdr1 (Figures 1A,B,D). These EHs possibly anchor Cdr1 to the plasma membrane and enable substrate translocation through the hydrophobic exit valve.

## 3 Site-directed and random mutagenesis of Cdr1

Site-directed mutagenesis has been used to investigate NBD residues involved in ATP-binding and hydrolysis (Jha et al., 2003; Jha et al., 2004; Rai et al., 2006) and TMD residues involved in the substrate transport and inhibition of Cdr1 (Shukla et al., 2003; Shukla et al., 2004; Gao et al., 2006; Rawal et al., 2013). By replacing seven serine or threonine residues with alanine and glutamate, this strategy helped confirm that the N-terminal extension of Cdr1 is phosphorylated in *C. albicans*, and that this modification is important for Cdr1 function (Tsao et al., 2016). Alanine scanning mutagenesis also elucidated the IL–NBD interface and provided valuable insights into how certain Cdr1 NBD motifs are involved in ATP binding (Shah et al., 2015; Rawal et al., 2016; Vishwakarma et al., 2019). Other studies have shown that even non-synonymous nucleotide substitutions in LD2 can cause increased drug resistance due to the increased mRNA stability of *PDR5* transcripts (Rahman et al., 2020).

UV-, hydroxylamine-induced, or error-prone random PCR, mutagenesis coupled with drug resistance screening, is another approach to identify mutations underlying transporter dysfunction which has been applied to Pdr5 (Egner et al., 1998; Tutulan-Cunita et al., 2005; de Thozée et al., 2007; Chen et al., 2014). The key mutation, S558Y (A548 in Cdr1), in TMS2 of Pdr5 was also discovered using this approach (Sauna et al., 2008).

## 4 Discussion of the use of drug resistance-based suppressor screening strategies

### 4.1 Identification of interactions of Cdr1 with inhibitors

Drug resistance–based suppressor screening enables unbiased identification of gain-of-function Cdr1 mutations. This likely mirrors the spontaneously arising resistance-associated Cdr1 mutations found in clinical isolates. The *S. cerevisiae* host strains AD1-8u^−^ (Decottignies et al., 1998), AD∆ (Lamping et al., 2007), and AD∆∆ (Sagatova et al., 2015) are particularly well suited for suppressor screening because they are drug hypersensitive due to the deletion of seven major efflux pumps, and the gain-of-function transcription factor *PDR1-3* enables exceptionally high membrane protein expression levels (Lamping et al., 2007). In addition, *S. cerevisiae* strains are generally recognized as safe (GRAS) and so restoring efflux pump function from pathogenic organisms expressed in *S. cerevisiae* is unlikely to be classified as gain-of-function research of concern (GOFROC) (Casadevall et al., 2024). Overexpression of Cdr1 in drug hypersusceptible hosts enables the application of strong positive selection pressure, which greatly enriches for intragenic mutations, while mutations outside *CDR1* are rarely (<1–5%) observed. This approach offers important mechanistic insights into transporter-inhibitor interactions.

Niimi et al. developed a D-octapeptide derivative, RC21-v3, that selectively inhibited Cdr1 and sensitized a *S. cerevisiae* strain overexpressing Cdr1 to fluconazole. To map the target sites of RC21-v3, they screened for spontaneous intragenic suppressor mutations that permitted growth in the presence of RC21-v3 and high, growth-inhibitory concentrations of fluconazole (Niimi et al., 2012). Each of the twelve suppressor clones contained one nucleotide substitution causing one amino acid change in Cdr1. Mutations clustered in the ECD (Q714K, Q1445K) and near the extracellular ends of TMS2 (G547R), TMS5 (V674F), and TMS8 (Q1226K/R) (Figures 1B,E; Table 1). A single positive charge was sufficient to disrupt RC21-v3 binding, indicating charge-based interference. Importantly, some mutations were found multiple times indicating saturation of spontaneous suppressor mutations (Table 1), thus mapping the extent of RC21-v3 interaction with Cdr1. Further experiments showed that RC21-v3 most likely stabilizes the inward-open conformation of Cdr1 (Niimi et al., 2022). Thus, RC21-v3 may serve as a tool to immobilize Cdr1 in the inward-open conformation and to obtain more structural information about the substrate exit region of Cdr1.

**Table 1:** Description of 121 suppressor mutations (single letter code) identified among 48 residues mutated in 12 separate Cdr1 suppressor screens.

Protein domain	Cdr1 residue	Mutations enabling FLC (*) or CHX (**) transport in the presence of various types of efflux pump inhibitors						Mutations recovering FLC transport of the indicated Cys-less Cdr1 variant			Mutations recovering FLC (*), ITC (**), or R6G (***) transport of Cdr1-E704 and -G521 variants		
		*RC21	*BEA	*ENI	*MIL	*FK	**FK	N	IC	EC	*K704	**RHFW521	***HYFW521
TMS1	D511											V	
	G521^a^		SCV	RV	R			R		A		S_2_C_2_PL	
	I528						T						
EL1	N535					I							
	T539						I						
	T540					I_4_							
	S542					L_2_P_4_							
	Y544					F							
TMS2	R546					T_4_						G	G
	G547	R				R							
	A548												G
	A549											V_2_	
EL2	V616						F						
TMS4	M639			I	I_3_								
IL2	V649											F	
TMS5	T658											P	
	L664			I									
	L665			S									
	V668		D	I									
HL1	G672												RA
	F673						L						
	V674	F_2_											
PDR-B1	V693							L					
	E704										NQE_5_		
EL3	C712					S_2_							S
	A713				P_3_	P_4_							
	Q714	K_2_				P							
	Y749						S						
	A753						P						
TMS6	L782											F	
NBD2	C1106								I_2_C_2_				
TMS7	A1207							T		VT	T_6_		
TMS8	L1225											I	
	Q1226	K_5_R											
	F1235			V		C_3_							
	P1238						Q						
	N1240					D							
TMS9	G1288						C						
	W1295					S							
TMS10	V1322					GA_3_							
TMS11	A1347						V						
	T1351						I						
	T1355				N								
	M1356			I									
HL2	A1365						D						
PDR-B2	F1383						LI						
	Q1388					P							
EL6	Q1445	K											
^b^Total/_#residues_		12_5_	4_2_	8_7_	8_4_	34_14_	13_12_	3_3_	4_1_	3_2_	13_2_	14_8_	5_4_
^c^Saturation level		2.4	2.0	1.1	2.0	2.4	1.1	1.0	4.0	1.5	6.5	1.8	1.3

FLC, fluconazole; CHX, cycloheximide; ITC, itraconazole; R6G, rhodamine 6G; BEA, beauvericin; ENI, enniatin B; MIL, milbemycin α25; FK, FK506 (also known as tacrolimus); N, Cdr1 with all N-terminal cysteines replaced with serine or alanine; IC, Cdr1 with all intracellular cysteines replaced with serine and alanine; EC, Cdr1 with the six extracellular cysteines replaced with serine.

a SCV in the second column indicates that in the search for BEA+FLC resistant mutants, three were mutated in G521 leading to S, C and V substitutions. S_2_C_2_PL in the second last column means that six of the Cdr1-R-, -H-, -F-, or -W521 variants secondary site suppressor mutants selected under ITC pressure were variants of the same residue, with substitutions to S and C occurring twice and to P and L once each.

b The total number of independently isolated suppressor mutants and the number of mutated residues. In the first column, “12_5_” indicates that 12 suppressor mutants were isolated under RC21 + FLC selection pressure, and these mutations were mapped to 5 positions within Cdr1.

c The saturation level is the total number of suppressor mutations divided by the total number of residues identified in the various suppressor screens.

Similar suppressor-based screening strategies have been used to identify key residues and elucidate their role in the inhibition of Cdr1 pump function by FK506, milbemycin α25, enniatin B, and beauvericin (Figures 1A,B,E). For FK506, two major hotspots emerged: (i) the EL3-TMD1 contact region with 24 suppressor mutations in N535, T540 and S542 (EL1), Y544, R546 and G547 (TMS2), and C712, A713 and Q714 (EL3); and (ii) a central TMS8-TMS10 contact region with 7 mutations in F1235 (TMS8) and V1322 (TMS10; Table 1; Figure 1A) (Tanabe et al., 2019). Seventeen of the twenty Cdr1 suppressor mutations whose FLC transport became resistant to the broad-spectrum fungal efflux pump inhibitors milbemycin α25, enniatin B, or beauvericin were in just eight residues (Table 1; Figure 1B): G521 (TMS1), M639 (TMS4), L664, L665, and V668 (TMS5), F1235 (TMS8) and T1355 and M1356 (TMS11). The remaining three milbemycin α25 suppressor mutations (A713P) were the same as the four A713P-FK506 resistance hotspot 1 suppressor mutations (Table 1) (Niimi et al., 2022).

An effective efflux pump inhibitor is not susceptible to the development of drug resistance. Based on this criterion beauvericin was the most promising and FK506 the least promising Cdr1 efflux-pump inhibitor. Despite extensive efforts, we could only isolate four beauvericin suppressor mutants with mutations in just two residues (i.e., 4/2; Table 1). Mutations in far more Cdr1 residues relieved FK506 inhibition of fluconazole (34/14) and cycloheximide (13/12) transport (Table 1).

Notably, G521, located centrally in TMS1, was a recurrent inhibitor resistance hotspot (Figures 1B,C). The G521R variant exhibited a substrate transport profile opposite to the G521 parent, with significantly reduced ability to transport large substrates and minimal impact on small substrates, suggesting that G521 may play a key role as Cdr1 gate-keeper (Niimi et al., 2022). The importance of this residue was also highlighted in another study by exposing Cdr1 expressing cells to high, toxic, concentrations of Snq2-specific efflux pump substrates. The isolation of Cdr1-G521D/S gain-of-function variants demonstrated a close association of G521 with substrate specificity (Kolaczkowski et al., 2013). Furthermore, screening with fluconazole+FK506 or cycloheximide+FK506 revealed inhibitor resistant suppressor mutations in similar TMD regions but each substrate/inhibitor pair induced mutations in a unique set of residues (Table 1), suggesting that substrate binding modulates inhibitor sensitivity (Tanabe et al., 2019).

### 4.2 Identification of important contact points within Cdr1

The suppressor screening strategy also enables the identification of secondary site mutations that can restore the function of defective Cdr1 variants. To rescue the function of the severely impaired (32-fold reduced fluconazole resistance) Cdr1 variant (N) with all nine N-terminal cysteines replaced by serine or alanine, strong positive selection pressure (~10 × MIC_FLC_) identified G521R, V693L, and A1207T mutations that were able to recover the efflux pump function of this variant (Table 1; Figure 1E) (Madani et al., 2021). A similar screen of cells expressing Cdr1 with all sixteen intracellular cysteines (IC) replaced with serine or alanine identified four mutations, all of the same mutated cysteine residue C1106S, converting S1106 to either the wild-type residue C1106 or to I1106 (Table 1). This revealed C1106 as a critically important NBD contact at the cytosolic apex of Cdr1. The study also identified C1106 as part of another important ABCG transporter motif (GX_2-3_CPX_3_NPXD/E) (Figure 1A) that was critically important for correct folding and trafficking of Cdr1 (Madani et al., 2021) and human ABCG1 (Wang et al., 2013). Finally, an attempt to rescue the inactive extracellular cysteine-less (EC) Cdr1 variant (Madani et al., 2021) identified once again mutations in G521 and A1207 (Table 1).

Site-directed mutagenesis of G521 helped confirm its gating function. A gradual increase in the side chain size caused a reduction in the transport of large substrates but the transport of smaller substrates such as cerulenin or cycloheximide increased slightly. To explore how these G521 variants could adapt to restore transport of large substrates, we screened for Cdr1-W/F/Y/H/R521 gain-of-function suppressor mutants that were able to grow in the presence of high (4–10 × MICs) rhodamine 6G or itraconazole concentrations. This search revealed key contact residues involved in the rigid body motion of the TMDs, clustered near the cytosolic membrane bilayer boundary or near the hydrophobic exit valve (unpublished data). There was a notable bias in the distribution of suppressor mutations that depended on the screening agent. Prominent among the rhodamine 6G suppressor mutants were mutations in R546 and A548 at the top of TMS2, G672 of the hydrophobic loop 1 (HL1) at the top of TMS5 and C712 of EL3. The conserved RGAA (R546-A549) motif of fungal PDR transporters (Lamping et al., 2010) at the top of TMS2 is in direct contact with the hydrophobic exit valve and E704 at the C-terminus of EH1 (Figures 1A,D).

Charge-reversal of the conserved glutamate residue, E704 inactivated the Cdr1-K704 variant. Only variants with N704, Q704, or E704, or an A1207T mutation could restore its transport function (Table 1). The N704 and Q704 variants were isolated under medium fluconazole selection pressure. However, increasing the selection pressure with higher fluconazole concentrations identified only two possible suppressor mutations: K704E (5) and A1207T (6). This study revealed A1207, located centrally in TMS7 and in close contact with TMS5 at the rear of the transporter, as a critically important pivot for the large conformational changes experienced by the TMDs during substrate translocation (Figures 1A,C).

The strategy of searching for secondary site suppressor mutants has also played a profound role in elucidating the signaling interface between the NBDs and TMDs (Shah et al., 2015), and in highlighting the functional relevance of the non-catalytic NBS (Banerjee et al., 2018; Banerjee et al., 2020). Drug pressure screening of Cdr1 variants with IL or TMD mutations identified secondary site mutations in the NBDs, underscoring the importance of long-distance intramolecular communication within this transporter (Banerjee et al., 2021). Intriguingly, screening of the I574A variant revealed distinct responses to fluconazole and ketoconazole: fluconazole only induced reverting A574 to wild-type (i.e., A574I), whereas all ketoconazole suppressors carried a secondary R935T mutation. This further highlighted the substrate-specific conformational adaptations of Cdr1.

### 4.3 Analysis of *Saccharomyces cerevisiae* Pdr5

Screening for suppressor mutants has also proven effective with cells overexpressing Pdr5. Several key Pdr5 residues were found to be functionally equivalent to those in Cdr1. For instance, T550, T552, A723, and T1364 of Pdr5 gave rise to similar FK506 resistance suppressor mutations as their Cdr1 counterparts T540, S542, A713, and T1355 (Tanabe et al., 2019). The HL1 equivalent residues Cdr1-V674 and Pdr5-A684 both yielded inhibitor resistant suppressor mutations, i.e., to RC21-v3 (V674F) and FK506 (A684S), respectively (Niimi et al., 2012; Tanabe et al., 2019). Pdr5-S558 and the equivalent Cdr1-A548 residue are both important components of the hydrophobic exit valve. Pdr5-S558Y was identified in a random mutagenesis screen for mutants with severely impaired efflux pump function and Cdr1-A548G was identified as a suppressor mutation that could recover rhodamine 6G transport of the Cdr1-G521H gating mutant (Sauna et al., 2008; unpublished data). These comparisons highlight the structural and functional similarities between these two important efflux pumps.

In a series of studies, the screening of the defective S558Y (TMS2) Pdr5 variant with clotrimazole selection pressure identified the secondary site mutations N242K and E244G in NBD1 (Sauna et al., 2008; Ananthaswamy et al., 2010). While the single N242K variant was sensitive to cycloheximide, further screening of the N242K mutant under cycloheximide selection pressure revealed V656L as a second site suppressor mutation in IL2 (Downes et al., 2013). These findings helped define the IL–NBD interface and supported a cis rather than trans configuration of the NBD-TMD interface in Pdr5. Screening for survival of wild-type Pdr5 on a high concentration of cycloheximide identified A666G as a gain-of-function mutation with enhanced transport ability but unchanged ATPase activity, revealing Pdr5-A666 as a residue involved in promoting cooperative interactions between drug binding sites (Arya et al., 2019).

FK506 + fluconazole selection pressure identified FK506 resistance hotspots in Pdr5 that were either similar (hotspot 1) or different (hotspot 2) from those in Cdr1 (Tanabe et al., 2019). Two FK506 resistance hotspot 2 mutations of Pdr5 (F683L at the top of TMS5 and M1373T at the top of TMS11) were part of the hydrophobic exit gate. The F683L mutation helped demonstrate that F683, like S1368 at the top of TMS11, is involved in preventing substrate reflux in Pdr5 (Alhumaidi et al., 2022). Likewise, the exposure of a hypersusceptible *S. cerevisiae* strain to high, toxic, concentrations of beauvericin caused the emergence of suppressor mutants that had mutations in Pdr5 (three of five variants had mutations in G538C/R at the top of TMS1) that converted beauvericin from a Pdr5 inhibitor to a pump substrate (Shekhar-Guturja et al., 2016). These findings give an insight into what distinguishes an effective efflux pump inhibitor from an efflux pump substrate. Mutations in Pdr5 were also isolated in caffeine tolerance studies (Surmeli et al., 2019; Geck et al., 2024). These studies suggest that overexpression and/or modifying Pdr5 efflux pump function may be a more favorable resistance mechanism than altering the actual drug targets of toxic efflux pump substrates. This also explains why *PDR5* had to be deleted first before it was possible to identify the global regulator TORC1 kinase as the actual drug target of beauvericin in the beauvericin resistant suppressor screen (Shekhar-Guturja et al., 2016).

## 5 Conclusion

Over the past decade, advances in structural biology have facilitated the determination of high-resolution ABC transporter structures. When integrated with biochemical insights from approaches such as site-directed mutagenesis, these developments have greatly increased our understanding of Cdr1 and related fungal PDR transporters. Despite this progress, critical gaps remain in our structural knowledge of the transport cycle, and which amino acid interactions affect pump function. Drug resistance–based gain-of-function suppressor screens have emerged as a particularly powerful tool to identify inhibitor binding hotspots and mechanisms of adaptive resistance, offering valuable insights for further structural interrogation and rational inhibitor design. The latter approach remains a promising strategy for restoring antifungal efficacy against drug-resistant *Candida* strains. The optimization of macrocyclic small molecules like beauvericin, one of the most promising broad-spectrum lead inhibitors, could be achieved by maximizing their hydrophobic interactions with the large substrate binding pocket to lock PDR transporters in an inward-open conformation. The ECDs of fungal PDR transporters may be an even more tempting drug target for small molecule inhibitors like RC21-v3. These ECDs are easily accessible from the extracellular space, and their unique structure means that any drugs targeting these structures could be expected to exhibit fewer potential side-effects when used clinically. Furthermore, the development of small molecules that trap Cdr1 in defined conformations represents a useful approach with dual applications – as structural probes and as therapeutic adjuvants that lock Cdr1 in inactive configurations. Continued efforts to identify and optimize these compounds hold strong potential to improve clinical outcomes and aid in capturing elusive conformational states of efflux pumps during the transport cycle.

## Data Availability

The original contributions presented in the study are included in the article, further inquiries can be directed to the corresponding author.
